# MDRD or CKD-EPI study equations for estimating prevalence of stage 3 CKD in epidemiological studies: which difference? Is this difference relevant?

**DOI:** 10.1186/1471-2369-11-8

**Published:** 2010-06-01

**Authors:** Pierre Delanaye, Etienne Cavalier, Christophe Mariat, Nicolas Maillard, Jean-Marie Krzesinski

**Affiliations:** 1 Department of Nephrology-Dialysis-Transplantation, University of Liège, CHU Sart Tilman, Liège, Belgium; 2 Department of Clinical Chemistry, University of Liège, CHU Sart Tilman, Liège, Belgium; 3 Department of Nephrology, Dialysis and Transplantation, University of Saint-Etienne, Hopital Nord, Saint-Etienne, France

## Abstract

**Background:**

Prevalence of stage 3 chronic kidney disease (CKD) is increasing according to the NHANES study. Prevalence has been calculated using the MDRD study equation for estimating glomerular filtration rate (GFR). Recently, a new estimator based on creatinine, the CKD-EPI equation, has been proposed which is presumed to better perform in normal GFR ranges. The aim of the study was to measure the difference in prevalence of stage 3 CKD in a population using either the MDRD or the CKD-EPI study equations.

**Methods:**

CKD screening is organized in the Province of Liège, Belgium. On a voluntary basis, people aged between 45 and 75 years are invited to be screened. GFR is estimated by the MDRD study equation and by the "new" CKD-EPI equations.

**Results:**

The population screened consisted in 1992 people (47% of men). Mean serum creatinine was 0.86 ± 0.20 mg/dL. The prevalence of stage 3 CKD in this population using the MDRD or the CKD-EPI equations was 11.04 and 7.98%, respectively. The prevalence of stage 3 CKD is significantly higher with the MDRD study equation (p < 0,0012).

**Conclusions:**

Prevalence of stage 3 CKD varies strongly following the method used for estimating GFR, MDRD or CKD-EPI study equations. Such discrepancies are of importance and must be confirmed and explained by additional studies using GFR measured with a reference method.

## Background

Prevalence of end-stage renal failure is increasing in Western Countries [1] even if this fact has been recently questioned [2]. In this context, prevention of chronic kidney disease (CKD) is of importance [3]. The first step for efficient prevention is an early diagnosis. However, serum creatinine is of limited value for such a task, as it is classically known that creatinine will rise over normal values only when 50% of glomerular filtration rate (GFR) have already been lost [4,5]. This creatinine lack of sensitivity is especially due to its relationship with muscular mass and will be noted particularly in population with lower muscular mass, notably the older population [4,6]. Several authors have proposed creatinine-based equation to improve GFR estimation. The MDRD study equation is one of the most used for this purpose [7]. So, epidemiological data have shown that nearly 10% of the population in the USA has stage 3 CKD or worse, defined as estimated GFR lower than 60 mL/min/1.73 m^2 ^with the MDRD study equation [1]. However, we and others have underlined that MDRD equation is not accurate and especially not precise for the estimation of GFR in healthy population and, more obviously, when it is applied to normal creatinine values [2,8,9]. There are several good reasons to believe that MDRD study equation underestimates GFR (and thus overestimate CKD prevalence) in these populations with normal or near-normal creatinine values [2,10,11].

According to these limitations, Levey's group who is already at the origin of the MDRD study equation has built a new creatinine-based equation from a large sample of CKD and healthy subjects. This CKD-EPI study equation includes, in fact, four different equations following the sex and the ethnicity (Table 1) [12]. It seems thus interesting to evaluate the prevalence of CKD (defined as estimated GFR under 60 ml/min/1.73 m^2^) with the MDRD study equation on one hand and with the CKD-EPI equation on the other hand.

**Table 1:** Creatinine- (SCr; mg/dL) based equations for glomerular filtration rate (GFR) estimation.

4-variable MDRD Study equation
GFR (mL/min/1.73 m^2^) = 175 × SCr ^-1.154 ^× Age ^-0.203 ^× 0.742 (if woman) × 1.21 (if black)

CKD-EPI Study equation (white subjects)
If woman:
if creatinine < 0.7 mg/dL:
GFR (mL/min/1.73 m^2^) = 144 × SCr/0.7 ^-0.329 ^× 0.993^age^
if creatinine > 0.7 mg/dL:
GFR (mL/min/1.73 m^2^) = 144 × SCr/0.7 ^-1.209 ^× 0.993^age^
If man:
if creatinine < 0.9 mg/dL:
GFR (mL/min/1.73 m^2^) = 141 × SCr/0.9 ^-0.411 ^× 0.993^age^
if creatinine > 0.9 mg/dL:
GFR (mL/min/1.73 m^2^) = 141 × SCr/0.9 ^-1.209 ^× 0.993^age^

## Methods

This study has been approved by the Ethic Committee of our University Hospital. The Belgian number study is B70720071509. This prospective study was driven in the context of the CKD screening program organized by the Province of Liège's Health deputation. Province of Liège is one of the ten provinces in Belgium. Its area is 3862 km^2 ^and its population from 2005 data has been calculated to reach a total of 1.037.161 inhabitants. The CKD screening is organized by a medical bus that travels through the 84 communes of that Province. On a voluntary basis, people aged over 45 years are invited to be screened. All patients have been informed and have signed informed consent. Data have been anonymously analyzed. Blood samples and anthropometrical data are collected and a short interview is done. Between June 2008 and April 2009, frozen blood samples were sent to the Clinical Chemistry laboratory of the University of Liège where they were immediately thawed and assayed. Serum creatinine was measured by the IDMS traceable Jaffé method from Roche (compensated Jaffé, Roche Diagnostics, Mannheim, Germany) on Modular [13,14]. Our university laboratory is currently accredited against the ISO 15189 Standard. As our creatinine is IDMS traceable, the new "175" MDRD study equation was used [7].

All results are expressed as mean ± SD. We have calculated and compared the percentage of patients with stage 3 CKD or worse obtained with the two equations, but also the coefficients of correlation between the different equations. Agreement between equations to discriminate GFR over and less than 60 mL/min/1.73 m^2 ^has been evaluated by Kappa statistics.

The results of GFR estimated by the MDRD study and the CKD-EPI equations have also been compared by Bland and Altman analysis [15]. In these analyses, we have arbitrarily chosen the MDRD study equation results as the referent. Bias between equations was defined as the mean of the differences. The SD around the mean reflected the dispersion and the precision of the equations. P < 0.05 was considered as significant. We have also evaluated if difference between equations might be correlated to variables such as age, sex, creatinine or estimated GFR levels. We also repeated Bland and Altman analysis in subgroups according to sex and level of estimated GFR (over or below 60 ml/min/1.73 m^2^). All analyses were performed using MedCalc^® ^(MedCalc Software, Mariakerke, Belgium).

## Results

During the study period, 1992 people were screened (47% male and 53% women). Anthropometric, clinical and biological characteristics of the global population are shown in Table 2. By paired samples t-test, the estimated GFR by the MDRD and the CKD-EPI study equations were different from each other (p < 0.0001). Prevalence of stage 3 CKD when GFR was estimated by the MDRD equation study was 11.04% (n = 220). This prevalence was significantly and strongly higher than the prevalence obtained when the CKD-EPI study equation which was 7.98% (n = 159)(p = 0.0012). However, Kappa statistics showed very good agreement between the two equations (κ = 0.82).

**Table 2:** Anthropometrical and biological description of the population (n = 1992).

N = 1992	Mean	SD	Range
Age (years)	62	8	45-84

Weight (Kg)	75	16	37-156

Height (cm)	167	9	140-196

BMI (Kg/m^2^)	27	5	15-60

Creatinine (mg/dL)	0.86	0.2	0,4-2,7

MDRD study (mL/min/1.73 m^2^)	82	18	18-166

CKD-EPI study (mL/min/1.73 m^2^)	84	16	18-123

Data are expressed as mean ± SD.

Results given by the two equations were highly correlated (p < 0.0001)(r = 0,93).

**Figure 1:**
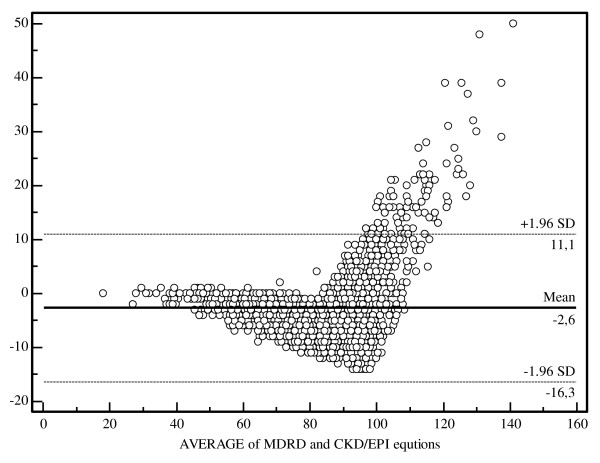
**Bland and Altman analysis between the MDRD study equation and the new CKD-EPI equations**. The continuous line represents the mean difference between estimated GFRs, whereas the dashed lines represent the limits of agreement (mean difference ± 2SD). All values are expressed in mL/min/1.73 m^2^.

The Bland and Altman analysis results are summarized in Figure 1. The mean difference between the MDRD study and the CKD-EPI equation was -2.6 ± 7 mL/min/1.73 m^2^.

By multinear regression analysis, difference between MDRD and CKD-EPI results was dependent upon age (the greater the age, the greater the difference, r = 0.4, p < 0.0001) and creatinine concentration (the lower the creatinine, the greater the difference, r = -0.2, p < 0.0001).

Two subgroups analyses were conducted following sex and estimated GFR. If we restricted analysis to estimated GFR under 60 mL/min/1.73 m^2 ^(with the MDRD study equation)(n = 220), the paired samples t-test still showed significant difference between the estimated GFR by the MDRD and the CKD-EPI study equations (p < 0.0001). Of course, this is still the case when results over 60 mL/min/1.73 m^2 ^were analyzed (n = 1772). The results of the Bland and Altman analyses were more interesting. Indeed, the mean difference between the MDRD study and the CKD-EPI equation was -2.3 ± 1.9 mL/min/1.73 m^2 ^and -2.7 ± 7.4 mL/min/1.73 m^2 ^when analysis concerned estimated GFR under and over 60 mL/min/1.73 m^2^, respectively. If the bias was equivalent in these two subgroups, deviation around the bias was significantly lower in the subgroup with estimated GFR less than 60 mL/min/1.73 m^2 ^compared to global population or the subgroup with GFR higher than 60 mL/min/1.73 m^2 ^(F-test, p < 0,001).

Anthropometrical and biological values were different between men and women, as it was illustrated in Table 3. Only, the mean results of the CKD-EPI equations were not different between men and women. Kappa statistics showed very good agreement between the two equations but agreement seemed slightly better for men than for women (κ = 0.92 and 0.76, respectively). The mean difference between the MDRD study and the CKD-EPI equation was -0.8 ± 7 mL/min/1.73 m^2 ^and -4.4 ± 6.5 mL/min/1.73 m^2 ^when analysis concerned men and women, respectively. The difference between these last results was highly significant (p < 0.0001). In the same way, prevalence of stage 3 CKD in men was 8.47% and 7.29% with the MDRD and the CKD-EPI equations, respectively (non significant difference). In women, prevalence of stage 3 CKD was highly different using either the MDRD or the CKD-EPI equations (13.31% *versus *8.59%, p = 0.0007).

**Table 3:** Anthropometrical and biological description of the population according to gender.

	Men (n = 933)	Women (n = 1059)	Difference
Age (years)	62 ± 8	61 ± 8	P = 0.0054

Weight (Kg)	83 ± 15	68 ± 13	P < 0.001

Height (cm)	174 ± 7	161 ± 6	P < 0.001

BMI (Kg/m^2^)	27 ± 5	26 ± 5	P < 0.001

Creatinine (mg/dL)	0.95 ± 0.2	0.78 ± 0.18	P < 0.001

MDRD study (mL/min/1.73 m^2^)	84 ± 18	79 ± 18	P < 0.001

CKD-EPI study (mL/min/1.73 m^2^)	85 ± 15	84 ± 16	NS

Data are expressed as mean ± SD.

## Discussion

Epidemiologic studies in different Western countries have recently shown that prevalence of CKD, defined as GFR under 60 mL/min/1.73 m^2^, is about 10% in the global population [1,16]. These data have been obtained with the MDRD study equation using a well calibrated serum creatinine [17,18]. However, the use of this equation is not free from criticisms. We, and others, have demonstrated that this equation tends to strongly underestimate GFR in healthy populations and, more generally, in patients with normal or near normal creatinine values [2,10,11,19]. Admitting this fact, the Levey's group has recently proposed a new equation which is thought to be especially better in the higher GFR range (over 60 ml/min/1.73 m^2^). In this view, the new CKD-EPI equations are different following the creatinine value (0.7 mg/dL for the women and 0.9 mg/dL for the men). This adaptation seems logical as relationship between GFR and creatinine is different in healthy as compared to CKD subjects. The better accuracy of the new equation is also certainly explained by the authors because the inclusion of healthy subjects in the equations development study (13% i.e. 694 healthy kidney donors). Logically, using the new CKD-EPI equations, the prevalence of stage 3 CKD in our population is significantly lower than if the MDRD study equation is used (7.98% *versus *11.04%). Over 1992 patients screened, 61 (3.06%) were classified as having stage 3 CKD with the MDRD study equation compared to the CKD-EPI study equation. These data are not negligible from an epidemiological point of view.

Differences between MDRD and CKD-EPI equations are especially larger for the highest estimated GFR values. If we arbitrary fix a limit of 60 ml/min/1.73 m^2 ^estimated by the MDRD study equations, differences between the two equations are significantly higher for the values over than below 60 ml/min/1.73 m^2 ^(-2.3 ± 1.9 *versus *-2.7 ± 7.4 ml/min/1.73 m^2^, respectively). These differences are logical according to differences between equations. More astonishing is the difference observed in subgroup analysis according to gender. The difference between the two equations seems more impressive in women than in men. This is not explained by the GFR level or age as women have higher mean GFR and are younger (see below for the age effect). This difference could be explained by the lower cut-off chosen in women for the CKD-EPI equations. As relationship between creatinine and GFR is exponential, it could be logical that consequences on difference results between the two equations are more important in women. Nevertheless, more studies in the future are needed to explain such discrepancies between estimators regarding to gender because, obviously, one of the two equations is especially inaccurate in women.

In their presenting CKD-EPI article, Levey *et al *have also compared prevalence of CKD in the NHANES study. If we consider the same definition of stage 3 CKD, these authors have found that prevalence of CKD in the NHANES study was 9.88% with the CKD-EPI equation and 10.82% with the MDRD study equations (Appendix Table 6 in [12]). Difference in prevalence is more impressive in our own study. One explanation could be the higher mean age in our population. Indeed, one major difference between the two equations is the "age factor". In the MDRD study equation, a constant exponent is applied to age (age^-0.203^) whereas age is an exponent in the CKD-EPI equation (0.993^age^). Indeed, we find a significant correlation between age and difference between MDRD and CKD-EPI results (regression coefficient of 0.39 in multiple regression analysis). However, even if the MDRD study equation's performance in older population remains controversial [20,21], the performance of the CKD-EPI equation in patients or subjects over 70 years old has not been studied (only 3% of patients between 70 and 75 years old in development data of the CKD-EPI equations study). Discrepancy between our results and NHANES study results could also be the result of potential anthropometrical differences between American and European populations.

The new CKD-EPI equation is certainly interesting and its performance will probably be better than the MDRD study equation in population free from renal disease. Yet, some limitations may be advanced. Firstly, the choice of the creatinine cut-off is logically different according to sex (0.9 mg/dL for men and 0.7 mg/dL for women). However, it would be also logical that equations vary with age as relationship between creatinine levels is also strongly influenced by age [4]. Secondly, we underline, once again, the lack of data regarding older patients (more than 70 years old) that are clearly underrepresented in the CKD-EPI study. Thirdly, we have recently criticized the way the new "IDMS" traceable MDRD and more precisely the factor 175 has been obtained to make results IDMS traceable [9,10]. This criticism is also valid for the CKD-EPI equation because serum creatinine measurements from the development data are coming from studies (MDRD and AASK for example) where creatinine had been measured with Jaffé methods. So, from our point of view, the factors used in the CKD-EPI equations (144 for women and 141 for men) are too low inducing a systematic overestimation of CKD prevalence. Lastly, the major criticism for the new CKD-EPI equations is its lack of advantage regarding its precision in estimating GFR. Indeed, in the Levey study, if for subjects with GFR over 60 ml/min/1.73 m^2^, the bias with measured GFR is improved when using the CKD-EPI equations as compared to the MDRD equation (median difference of 3.5 *versus *10.6 mL/min/1.73 m^2^), however, the precision of the CKD-EPI equation in the same range of GFR doesn't appear better (and even seems slightly worse) than those of the MDRD (precision is reflected by interquartile range for differences: 25.7 *versus *24.2 mL/min/1.73 m^2^, respectively) [12]. So, if GFR estimation by CKD-EPI equation has an improved systematic bias and accuracy, this equation does not improve the precision of the estimation. This is disappointing but not astonishing as bias is, by nature, systematic although precision is random and especially linked to the precision of the creatinine measurement. The latter is not improved in the CKD-EPI equation in comparison with the MDRD equation (as already mentioned, creatinine has been measured with Jaffé methods) [8,9].

There are some limitations to our study. First, the main limitation is linked to the fact that we have not measured GFR with a reference method. So, even if we have indirect arguments to affirm that MDRD study equations overestimate CKD prevalence in global populations, such an assertion can only be checked if a reference method GFR is used. Our data only underline potential strong discrepancies between results in epidemiological studies when either the MDRD or the CKD-EPI study equations are used. Epidemiological studies on renal function in the global population are still urgently waited but it represents heavy work. Second, our stage 3 CKD prevalence data are of interest only because they illustrate these discrepancies. As our population is clearly not representative of the Belgian population (because only volunteers are included), our stage 3 CKD prevalence results must not be considered for epidemiological considerations. For this reason, data regarding proteinuria or hypertension status that are lacking in this study are of relatively poor interest in our study. Third, we have no data on the ethnicity. As the ethnicity factor varies following the equations, this could be source of bias. However, in Belgium, Caucasians are, from far, the dominant ethnic group. Moreover, there is little doubt that differences observed in our study are not due to the ethnic factor (1.21 in the MDRD study equation and 1.16 in the CKD-EPI equations). Four, like in several epidemiological studies, our subjects have been tested only once, although definition of CKD *sensu stricto *implies that two or three testing have been undertaken over a three months period. Last, we have defined CKD as GFR less than 60 mL/min/1.73 m^2^. The definition of CKD is however subject of debate, notably in elderly population [2,22]. Whatever, our data do not permit to bring to close this debate.

## Conclusions

The present study has illustrated large discrepancies observed in the prevalence of stage 3 CKD in a population according to the method used for estimating GFR. These differences seem especially relevant in women, older and subjects with estimated GFR over 60 ml/min/1.73 m^2^. We have argument to disfavour MDRD study equations in epidemiological studies but it must not be concluded that the CKD-EPI equations is absolutely free from criticisms. For example, we have recently illustrated discrepancies in CKD prevalence using either the MDRD and the cystatin C-based equations [8,23]. We have also strong discrepancies between CKD prevalence using either CKD-EPI or the cystatine C-based equations (in our population, prevalence of stage 3 CKD with the Levey cystatin C-based equations (GFR = 76.7 × CC (mg/L) ^-1.19^) is as low as 1.1%, data not detailed). More studies are urgently needed to confirm and explain these discrepancies. Epidemiological population studies using GFR measurements with a reference method are, once again, urgently required.

## Abbreviations

CKD: chronic kidney disease; GFR: glomerular filtration rate; SCr: serum creatinine.

## Competing interests

The authors declare that they have no competing interests.

## Authors' contributions

PD is the principal investigator and the first author of this manuscript. EC is the Biochemist who has measured serum creatinine. CM and NM have analyzed the data. JMK has critically corrected the manuscript as the Chief of the Department of Nephrology. All authors have read and approved the final manuscript

## Pre-publication history

The pre-publication history for this paper can be accessed here:

http://www.biomedcentral.com/1471-2369/11/8/prepub
